# Roaring High and Low: Composition and Possible Functions of the Iberian Stag's Vocal Repertoire

**DOI:** 10.1371/journal.pone.0063841

**Published:** 2013-05-08

**Authors:** Daniela Passilongo, David Reby, Juan Carranza, Marco Apollonio

**Affiliations:** 1 Department of Science for Nature and Environmental Resources, University of Sassari, Sassari, Italy; 2 Mammal Vocal Communication and Cognition Research, School of Psychology, University of Sussex, Brighton, United Kingdom; 3 Ungulate Research Unit, Game and Fish Research Center, University of Córdoba, Córdoba, Spain; University of Milan, Italy

## Abstract

We provide a detailed description of the rutting vocalisations of free-ranging male Iberian deer (*Cervus elaphus hispanicus*, Hilzheimer 1909), a geographically isolated and morphologically differentiated subspecies of red deer *Cervus elaphus*. We combine spectrographic examinations, spectral analyses and automated classifications to identify different call types, and compare the composition of the vocal repertoire with that of other red deer subspecies.

Iberian stags give bouts of roars (and more rarely, short series of barks) that are typically composed of two different types of calls. Long Common Roars are mostly given at the beginning or at the end of the bout, and are characterised by a high fundamental frequency (F0) resulting in poorly defined formant frequencies but a relatively high amplitude. In contrast, Short Common Roars are typically given in the middle or at the end of the bout, and are characterised by a lower F0 resulting in relatively well defined vocal tract resonances, but low amplitude. While we did not identify entirely Harsh Roars (as described in the Scottish red deer subspecies (*Cervus elaphus scoticus*)), a small percentage of Long Common Roars contained segments of deterministic chaos.

We suggest that the evolution of two clearly distinct types of Common Roars may reflect divergent selection pressures favouring either vocal efficiency in high pitched roars or the communication of body size in low-pitched, high spectral density roars highlighting vocal tract resonances. The clear divergence of the Iberian red deer vocal repertoire from those of other documented European red deer populations reinforces the status of this geographical variant as a distinct subspecies.

## Introduction

Vertebrate vocal signals are characterised by a very strong acoustic diversity: vocalisations can differ considerably between closely related species [1] and, within species, between subspecies [2], [3] or even between geographically isolated populations [4]–[6]. The main factors affecting acoustic variation in vocal behaviour are functional (the acoustic structure of signals reflects the type of information they transmit) [7]–[9], phylogenetic (related species tend to have more similar vocal repertoires [10]–[12], though isolated populations can evolve very different signals) [13], [14] and environmental (the sound propagation properties of the environment in which species live affects their structure [15], [16], as predicted by the “acoustic adaptation hypothesis” [17]). As a consequence, very closely related populations evolving in different habitats can evolve vocalizations which serve the same overall function but which make very different use of the acoustic space [13], [17].

Most species use vocalisations to mediate inter- or intra-sexual interactions during the period of reproduction. Sexual calls typically function to signal the caller's presence [18], [19], its reproductive status [20] and/or its quality [21]–[23]. Consequently, selection pressures have favoured the evolution of species-specific signals with acoustic properties that either optimise their active space [24], facilitate localization accuracy [25] in the species' habitat or reliably encode information about the individual's physical quality and social status [26]–[29].

The mating calls of Eurasian polygynous deer provide a clear illustration of the variability that results from these evolutionary processes, with a high degree of diversity among species of the *Cervinae* [11] but also among geographic subspecies of red deer *Cervus elaphus* (Linnaeus, 1758) [30]. This species is divided in several subspecies (for a list see [31]), which are geographically isolated and morphologically differentiated [32], but all characterised by strong vocal activity of the males (stags) during the breeding season.

During the mating season, which normally takes place between August and October in the northern hemisphere, red deer stags may herd and defend groups of females (harems) [27] or compete for and defend territories where females are subsequently attracted [33]–[35]. Throughout this period stags are highly vocal, and give several different types of calls, directed towards females and/or male competitors [27].

While early investigations of loud-calling in polygynous deer focussed on the calling rate as an indicator of resource holding potential [18], [27], [36], more recent studies have focused on the acoustic structure of the calls [29], [30], [37]–40 and the function of their key acoustic components in the context of male competition [21] and female choice [19], [41]–[43]. These investigations have greatly benefited from the application of the “source-filter” theory of voice production [44], [45] to non- human vocal signals. According to this theory, the main components of the calls, the fundamental frequency (F0) and the vocal tract resonances (or formants) are produced independently [44]–[46]. The glottal wave is produced at the level of the source (the larynx), by the vibration of the vocal folds caused by the passage of air through the closed glottis. The rate of vocal fold vibration determines the fundamental frequency of the vocal signal and affects its perceived pitch, a highly distinctive and variable characteristic of mammal calls [42]. Several studies of mammalian species have shown that within species and sex classes, F0 is not a reliable index of body size [47], [48], suggesting that other physical or physiological factors may influence the variation of this relatively unconstrained and dynamic acoustic feature. Interestingly, F0 is also highly variable between red deer subspecies: Scottish red deer (*Cervus elaphus scoticus*) [30] have a mean F0 of around 112 Hz, which is considerably higher than the mean F0 of 30 Hz reported in the roars of smaller Corsican red deer (*Cervus elaphus corsicanus*) [49], in contradiction with the general prediction that larger animals should have larger and heavier vocal folds and thus produce lower frequencies [50], [51]. While consistent with the experimental demonstration that female Scottish red deer prefer high-pitched roars [41], this observation suggests that very different selection pressures may have affected the evolution of this feature in these very closely related taxa.

In the second stage of voice production, the glottal wave travels through the supra-laryngeal vocal tract, which acts as a filter, and shapes the frequency spectrum by superimposing vocal tract resonances, also called *formant* frequencies [44]. Formant frequencies, and their overall frequency spacing (also called formant dispersion [45]), are inversely correlated with the length of the vocal tract (the distance between larynx and lips or nostril) [45], and because the length of the vocal tract is normally constrained by body size, formant characteristics typically provide a reliable indication of body size to receivers [45], [49], [52]. Moreover, because they depend on the shape of the supra laryngeal vocal tract, formant frequencies also encode individual variation in many mammals species (rhesus macaques (*Macaca mulatta*): [45]; fallow deer (*Dama dama*): [38], [53]; red deer: [49], [54]). Scottish and Corsican red deer males have a descended larynx [37], and are able to drop their highly mobile larynx further down towards the sternum, which allows them to increase their vocal tract length (VTL) while they vocalize [28], [30], [37]. However, in Scottish red deer, the minimum formant spacing achieved when the vocal tract is fully extended is still an honest indicator of body size [28] and playback experiments have demonstrated that receivers of both sexes use this acoustic indicator of body size during the rut: while females prefer roars with lower formants indicating larger males [41], harem-holding males respond more aggressively to playbacks of roars where lower formants indicate larger, more threatening, males [21].

Finally, the composition of vocal repertoires varies between geographical subspecies of red deer: studies of male vocal behaviour in Scottish red deer have identified five distinct vocalizations, which vary in relation to their acoustic structure and their contexts of emission: *Common Roars*, *Harsh Roars*, *Grunt Roars*; and two kinds of barks: series of *Chase Barks* and single *Loud Barks* [30]. In contrast, only *Common Roars* and *Chase Barks* have been described in Corsican deer stags [49]. While Common Roars typically sound tonal and have a spectral structure characterised by well-defined harmonics, they can also contain noisy segments characterized by non-linear phenomena (subharmonics and deterministic chaos) [30]. The acoustic structure of Harsh Roars is similar to that of the noisiest segments of the Common Roars, with a poorly defined or absent fundamental frequency and harmonics. Harsh Roars are characterised by weaker formant modulation and the absence of a pronounced drop in formant frequencies at the beginning of the roar [30]. Grunt Roars are acoustically very similar to Harsh Roars but shorter and given in short series [30]. Finally Chase Barks are short, explosive calls typically given in series by red deer stags as they chase a hind or a subordinate male; while longer Loud Barks are single calls generally given by stags as they stand still [30].

Iberian red deer (*Cervus elaphus hispanicus*, Hilzheimer1909) inhabit the Iberian Peninsula and are currently geographically isolated from other red deer populations in Eurasia and Maghreb [55], [56]. The Iberian subspecies (125 kg [56], [57]) is similar in size to the Scottish subspecies (121 kg for the Rum population, [32]), but larger than the Corsican deer (88 kg [49]). As in other European subspecies, Iberian red deer males engage in very intense vocal activity during the mating season, which is assumed to function as a means of attracting females and/or threatening opponents [34], [35]. However, the vocal repertoire of the Iberian deer has not yet been systematically investigated. Indeed, while a recent comprehensive study of the anatomy and behaviour of vocal production in Iberian red deer males [57] reports three distinct groups of calls (Long Common Roars, Harsh Roars and Short Common/Grunt Roars), it does not systematically describe and quantify the acoustic variation within and between these call types.

Here, we combine spectrographic examinations, spectral analyses and unsupervised, automated classification techniques to systematically examine the qualitative and quantitative acoustic variation in the vocal signals of free-ranging male Iberian red deer, with the aim of identifying the different call types that compose the repertoire of this subspecies. We then discuss the potential function of these different call types and compare the Iberian vocal repertoire with that previously described in other subspecies.

## Materials and Methods

### Study Site and Population

The study area in Doñana National Park (Andalucía, Spain) included a western area with Mediterranean shrub land and an eastern area with a marsh (which was dry during the period of study), separated by a long narrow strip of land with meadows and rushes. The climate is typically Mediterranean with hot, dry summers and mild, wet winters. All fieldwork was carried out with the authorisation of the authorities of the National Park. The red deer rut in Doñana usually takes place between the 1^st^ and the 25^th^ of September [35]. During this period, males typically move from their home ranges to the area used by females and use either harem-holding or territorial tactics to monopolize females [33], [34], [58]. Data collection took place during the 2010 rutting season.

### Data Collection and Recording

Calls of adult males, individually identified from the size, shape and branching pattern of their antlers, were recorded around dusk (corresponding to the period of maximum activity for red deer in South Western Spain [59]), from either fixed positions or car transects. Vocalizations were captured with a Sennheiser directional microphone fitted with a windshield (ME67 head with K6 power module – frequency response: 50–20,000 Hz) and saved on a hand-held Sony PCM D-50M digital recorder, in uncompressed “.wav” format with a 44,100 Hz sampling rate and 16 bits amplitude resolution. Vocalizations with high levels of background noise were excluded from the analyses.

For the purpose of the repertoire classification, we extracted and analysed 115 bouts (334 roars) recorded from fixed positions (at distances ranging from 70 to 200 m) from 13 adult males. For the purpose of the detailed acoustic comparisons between call types and between individuals, we extracted and analysed a smaller set of 82 bouts (144 calls) from 6 males recorded at closer distances (ranging from 49 m to 106 m) during car transects. Recording distances were measures using a Leica Range Master CRF 900 7×24 telemeter.

### Acoustic analysis

All analyses were performed on a HP Compaq nx7400 laptop computer with a SoundMAX integrated Digital HD Audio soundcard using Praat version 5.2.13 DSP package for Windows [60]. The spectral composition of each vocalisation was examined using narrow-band spectrogram (window length = 0.03 s; time step = 0.01 s; frequency step = 250; frequency resolution = 20 Hz; Gaussian window shape). The position of each call within the bout (Posbout) was assigned as: first, intermediate, last or single. A total of 20 variables were analyzed from calls and bouts (Table 1).

**Table 1:** List of acoustic variables used in the cluster analysis.

Duration	**Call duration (s)**	
MaxF0	**Maximum fundamental frequency (Hz)**	
MeanF0	**Mean fundamental frequency (Hz)**	
MinF0	**Minimum fundamental frequency(Hz)**	
RangeF0	**Difference between maximum and minimum frequencies (Hz)**	
PosBout	**Position within bout: First-Intermediate-Last-Single**	
DC/No DC	**Occurrence of deterministic chaos**	
F_1_, F2,..., F_8_	**Centre frequency of the first 8 formants (Hz)**	
ΔF	**Formant dispersion (Hz)**	
eVTL	**Estimated vocal tract length (cm)**	
dB		**Relative intensity of the call (dB)**
DurTot	**Duration of the bout (s)**	
Calls/Bout	**Number of units into the bout**	

#### Source-related parameters

Pitch values for each call were extracted using a forward cross-correlation [to pitch (cc) command] algorithm in Praat. The time step in the analysis was 0.03 s and the specified expected values for limits of pitch ranged between 30 and 300 Hz. Pitch variables included in the analysis were: mean (MeanF0); minimum (MinF0); maximum (MaxF0) and range (RangeF0). Duration of the calls (Duration) was also calculated (Table 1). The presence of Deterministic Chaos (DC), characterized by widespread energy and weak harmonic structure [53], was also investigated using visual inspection of the narrow-band spectrograms.

#### Filter-related parameters

**Figure 1:**
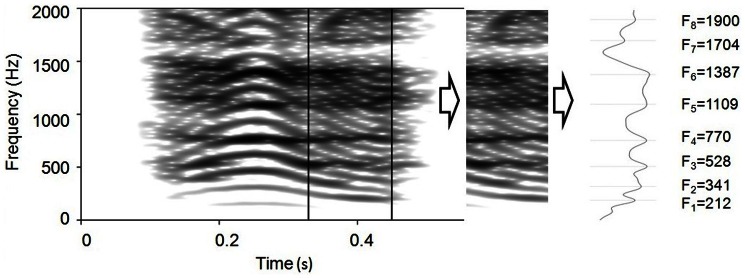
Extraction of the minimum frequency of individual formants in a Iberian deer roar. A spectrum was computed on the section of the spectrogram where individual formants reach their lowest frequencies. Cepstral smoothing was applied to the spectrum in order to remove the periodicity of the source (F0), thereby highlighting the effect of the filter.

Vocal tract resonances were only measured in the subset of calls recorded at shorter distances (87 Long Common Roars and 57 Short Common Roars). Formants' centre frequencies were measured on short segments located towards the end of the calls where the fundamental frequency (average: 126 Hz measured in a random sample of 20 calls from 4 animals) was sufficiently low to highlight the resonant properties of the vocal tract. This section was also characterized by close-to-minimal formant frequencies [28], [30], achieved as vocal tract is fully extended. A cepstral smoothing filter was applied to the spectrum in order to remove the contribution of the source periodicity (F0) from the frequency spectrum ([cepstral smoothing] command in Praat), thereby highlighting the effect of the filter (Figure 1). A filter bandwidth ranging between 120 and 150 Hz was used following visual inspection of the spectrum. The values of the first 8 formants were extracted from the resulting smoothed spectrum using the command [to formants] in Praat. We calculated formant dispersion (ΔF) and estimated Vocal Tract Length (eVTL) following the method outlined in [28], [30]. According to this method, when the supra-laryngeal vocal tract is approximated as a straight uniform tube, closed at one end (the glottis) and opened at the other end (the mouth), the spacing between any two successive formants can be approximated as a constant. Formant frequencies (*Fi*) can be plotted as a function of their predicted spacing *Fi* = ΔF(2*i*−1)/2, and the slope of the linear regression *Fi* = ΔF*(*x*), where *x* = (2*i*−1)/2, can be used as an estimate of formant dispersion ΔF. We can then estimate the apparent vocal tract length as eVTL = c/(2*ΔF), where *c* is the speed of sound in air (350 m/s) [28], [45], [46], [61]. All formants values were verified by visual inspection of narrow band spectrograms.

#### Intensity

The average energy intensity (dB) of each call was extracted using the [get intensity] command in Praat for within-bout comparison.

### Statistical analysis

All analyses were computed with R 2.14.0 statistical software (R Development Core Team 2010) [62]. All values are reported as mean ± standard error (SE).

In order to classify the vocal repertoire, we used explorative cluster analysis to identify relatively homogeneous groups of cases. Because one variable (deterministic chaos) was binary, analysis was performed on the dissimilarity matrix rather than on the original dataset [54]. A series of agglomerative hierarchical clustering was performed with the “AGNES” (AGglomerative NESting) function in the library “cluster” of R, changing the number of input variables (from 2 to 6) until the highest silhouette value was reached. Ward's method was used to link groups to each other, and the Euclidean squared distance was chosen as a measure of similarity. Silhouette information was computed as a means of interpreting and validating clusters of data [63]. Silhouette plots for different cluster solutions (from 2 to 8 clusters) were compared and the cluster with the highest values was chosen as the best solution.

The acoustic variables of the call types identified in our repertoire classification were subsequently compared using linear mixed models (LMM) (“lme” command of “nlme” package for R) with bout nested within individual as random factors and call type as fixed factor, to the subsequent acoustic dependent variables: Duration, MaxF0, MeanF0, MinF0 and RangeF0. In order to compare the means of the acoustic variables between the call types we performed simultaneous tests (Tukey contrasts) for linear hypotheses (“glht” function in the “multcomp” function in R).

We further compared the detailed acoustic structure (including relative amplitude and vocal tract resonances) of the two main types of roars identified in our repertoire classification (Long Common Roars and Short Common Roars) using calls from the short distance recordings data set. To do this we applied a linear mixed models (with bout nested within individual as random factors and call type as the fixed factor) to the following variables: Duration, amplitude (dB), source-related variables (MaxF0, MeanF0, MinF0, RangeF0) and filter-related variables (F1,F2,...,F8, ΔF, eVTL). Long Common Roar with DC and Chase Bark were not included in this analysis because we did not have a sufficient sample across different individuals.

Finally, in order to estimate inter-individual differences in the acoustic structure of male vocalizations we applied a discriminant function analysis (DFA) (“lda” function in the “MASS” library of R). DFA was performed on the acoustic variables characterising the first Long Common Roar of 79 bouts from the 6 individuals recorded at short distances. The identity of the stag was the group identifier and the acoustic variables (transformed to *z*-scores) were the discriminant variables. We performed a leave-one-out cross-validation procedure, and the percentage of correct classification was interpreted against the chance percentage expected for 6 males (16.6%).

## Results

### Call classification

**Figure 2:**
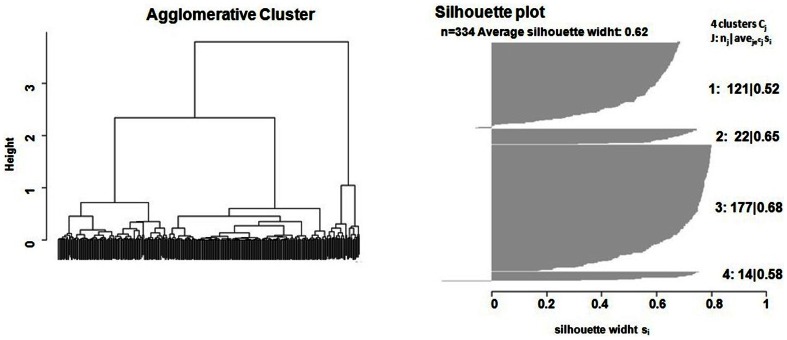
Cluster tree and silhouette plot. Cluster analysis was used to detect the presence of relatively homogeneous groups of calls. Silhouette Information was computed as a method of cluster interpretation and validation; the highest average silhouette classification score (0.62) was achieved by a four-groups solution based on DC, duration and maximum fundamental frequencies as input variables.

In the exploratory cluster analysis, the highest average silhouette classification score (0.62) was achieved by a four-group solution (Figure 2) based on presence of deterministic chaos, duration and maximum fundamental frequencies. Single silhouette values were 0.52 for the first group (N = 121); 0.65 for the second group (N = 22), 0.68 for the third group (N = 177), while a small number of calls (N = 14) were classified in a fourth group (silhouette score = 0.58) (Figure 2). The calls contained in the 1^st^ group, identified as *Long Common Roar*s (LCR) were roars characterised by a long duration (1.83±0.12 s), a high max F0 (207.2±9.0 Hz), and the absence of DC. The mean F0 measured in these calls (180.6±8.9 Hz) confirms the observations of Frey and colleagues [57]: 186.0±27.0 Hz.

The calls contained in the 2nd group were identified as *Long Common Roars with deterministic chaos* (LCRDC), and were characterised by the presence of segments of DC as well as a long duration (2.11±0.14s) and a high max F0 (220.3±10.8 Hz). The calls contained in the third group were identified as *Short Common Roars* (SCR) and were characterised by a short duration (0.44±0.12 s), a relatively low max F0 (142.4±9.0 Hz) and the absence of DC. Finally the fourth group contained series of short barks (*Chase Bark*-CB), and were characterised by the presence of DC, a low max F0 (133.9±10.2 Hz) and a short duration (0.27±0.12 s).

**Figure 3:**
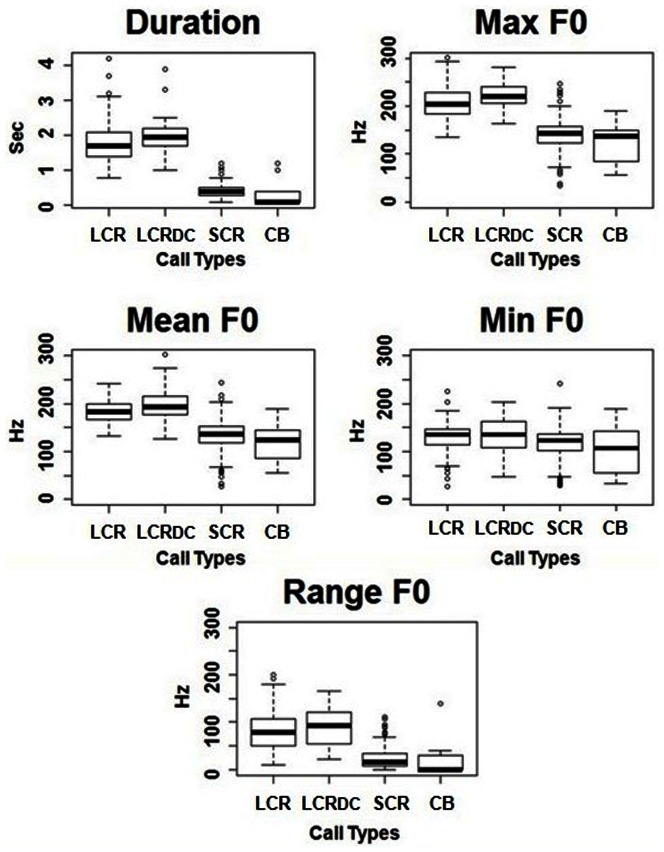
Box plots illustrating variation of the acoustic variables between call types. LCR: Long Common Roar; LCR_DC_: Long Common Roar with deterministic chaos; SCR: Short Common Roar; CB: Chase Bark.

The statistics associated with the LMM used to compare the acoustic variables between call types are reported in Table 2. There were highly significant differences between call types for all of the analysed variables (Table 2 and Figure 3), including among those not included in the explorative cluster analysis. Pairwise comparisons (reported in Table S1) show that all the call types significantly differ for all variables, with the exception of the Short Common Roar and Chase Bark, which only differ in the presence of DC in the Chase Bark, and the Long Common Roars with and without DC, which only differ in their duration (t = 3.03, *p* = 0.01).

**Table 2:** Comparison of acoustic variables between the four call types identified in the automated cluster analysis.

	LCR	LCRDC	SCR	CB	F(3,161)	*p*
**Duration**	1.83±0.12	2.110.14	0.44±0.12	0.27±0.12	347.46	<0.0001
**MaxF0**	207.2±9.0	220.3±10.8	142.4±9.0	133.9±10.2	137.35	<0.0001
**MeanF0**	180.6±8.9	196.3±10.5	133.1±8.9	120.0±9.9	87.86	<0.0001
**MinF0**	121.2±10.1	127.9±11.8	112.6±10.0	91.2±11.3	5.09	0.002
**RangeF0**	85.6±9.1	93.0±11.0	29.4±9.2	38.3±9.9	94.09	<0.0001

Estimated marginal means ± SE, F and *p* values for all the measured variables across the four call types. LCR: Long Common Roar, LCRDC: Long common roars with deterministic chaos; SCR: Short Common Roar; CB: Chase Bark.

**Figure 4:**
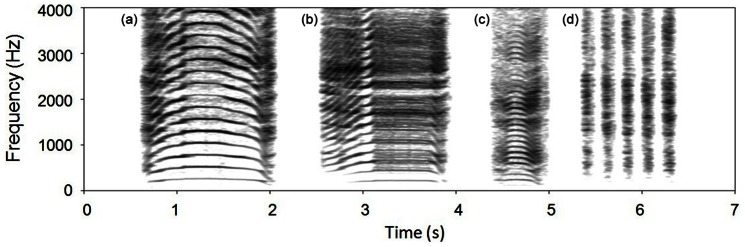
Spectrograms illustrating the acoustic structure of the four call types. Long Common Roar (a), Long Common Roar with deterministic chaos (b), Short Common Roar (c) and series of Chase Barks (d).

Despite the relatively small number of calls belonging to the Long Common Roar with DC and Chase Bark categories (respectively 22 and 14 in a total sample of 334 recordings), out of the 13 individuals recorded, 9 emitted LCRDC and 4 emitted CB. Spectrograms of representative examples of each of the identified call types are presented in Figure 4.

### Bout structure

Call sequences or “bouts” (N = 115) contained a variable number of calls (1–12), with a mean of 3.1 calls per bout (Calls/Bout). More than 40% of the bouts only included one call. Bout duration (DurTot) ranged from 0.85 s to 11.56 s with a mean of 4.36±2.57 s. Call types were not randomly distributed within bouts: while 68 out of 76 bouts started with a LCR or a LCRDC, SCR and CB were mostly emitted in the middle of the bouts (Table 3).

**Table 3:** Distribution of call types as a function of position within bouts.

		Position within bout				
Call Type		First	Intermediate	Last	Single	Total
**LCR**	**Count(%)**	**58(48%)**	20(16%)	21(17%)	22(18%)	121
**LCRDC**		**10(45%)**	2(9%)	4(18%)	6(27%)	22
**SCR**		7(4%)	**126(71%)**	41(23%)	3(2%)	177
**CB**		1(7%)	**9(64%)**	4(29%)	0(0%)	14

The most frequent position for each call type is highlighted in bold. LCR: Long Common Roar, LCRDC: Long common roars with deterministic chaos; SCR: Short Common Roar; CB: Chase Bark.

### Call comparison and formant frequencies

LMM comparisons between the two main vocalizations, LCR and SCR, confirmed the means and differences in pitch-related parameters and duration (Table 4). While eVTL and ΔF did not differ significantly between the two calls (Table 5), comparison of the intensity suggests that LCR are generally 10dB louder than SCR (Table 4). While overall formant frequency values (Table 5) were similar to those reported in the previous study by Frey and colleagues [57], formant dispersion (ΔF), calculated as minimum values of the eight formants by linear regression, was 247.1±1.7 Hz and the average estimated vocal tract length during roaring (eVTL) was 71.3±0.5 cm which is slightly lower than previously reported by Frey and colleagues [57].

**Table 4:** Comparison of source-related acoustic characteristics between Long Common Roars (n = 87) and Short Common Roars (n = 57).

	LCR(n = 87)	SCR(n = 57)		
	Mean±SE	Mean±SE	F(1,61)	*p*
**Dur**	1.90±0.05	0.52±0.07	405.44	<0.0001
**MaxF0**	202.6±5.5	144.8±4.9	137.01	<0.0001
**MeanF0**	172.2±5.3	121.3±3.1	262.70	<0.0001
**MinF0**	93.9±4.3	78.8±3.1	22.69	<0.0001
**RangeF0**	109.5±3.8	65.4±3.5	62.90	<0.0001
**dB**	70.1±1.7	60.7±0.8	123.73	<0.0001

Estimated marginal means ± SE, F and *p* values for Duration, source-related variables and relative intensity (dB) across LCR and SCR in the subset of calls recorded at shorter distances. LCR: Long Common Roar, SCR: Short Common Roar.

**Table 5:** Comparison of filter-related acoustic characteristics between Long Common Roars (n = 87) and Short Common Roars (n = 57).

	LCR(n = 87)	SCR(n = 57)		
	Mean±SE	Mean±SE	F(1,61)	*p*
**F1**	203.0±2.5	219.7±4.5	12.77	<0.001
**F2**	401.7±4.8	415.9±10.5	2.41	0.125
**F3**	623.7±6.4	622.8±13.1	0.01	0.957
**F4**	787.7±10.7	828.7±17.0	4.72	0.033
**F5**	1073.6±11.6	1112.9±15.1	5.09	0.027
**F6**	1370.0±11.7	1366.4±15.2	0.03	0.857
**F7**	1625.6±16.9	1605.2±21.9	0.59	0.441
**F8**	1875.9±20.8	1844.7±25.6	0.43	0.511
**ΔF**	247.0±2.2	247.1±2.8	0.03	0.851
**eVTL**	71.2±0.6	71.3±0.7	0.01	0.898

Estimated marginal means ± SE, F and *p* values. LCR: Long Common Roar, SCR: Short Common Roar.

### Individual variation

The DFA enabled the correct re-classification of 61.7% of the vocalisations (as opposed to the chance percentage = 16.6%). This percentage fell to 38.2% when a more conservative leave-one-out cross validation was applied. The first discriminant function accounted for about 72% of the variance and correlated highly with Mean F0 and F2. The second discriminant function accounted for about 14% of the variance and correlated with MeanF0, MaxF0, F1, F4, F5, F6 and F8; the third discriminant function was mostly related to F3, F5 and F6, and explained 6% of the inter individuals variance whilst the other two discriminant functions only accounted for 8% of the total variability (Table 6).

**Table 6:** Structure matrix of the discriminant function analysis characterizing individual differences.

	Function				
Variables	1	2	3	4	5
**Duration**	0.31	0.51	0.33	0.45	0.10
**MaxF0**		**−1.27**	0.26	0.29	
**MeanF0**	**−0.81**	**1.04**		**−0.72**	0.45
**MinF0**			0.45	0.51	**0.66**
**F1**	−0.55	−**0.84**	0.22	−0.31	
**F2**	**1.11**	0.46	−0.51	0.28	0.58
**F3**	0.34		**0.82**	−**0.64**	0.24
**F4**		−**0.80**	−0.58	0.29	0.70
**F5**	−0.35	**1.03**	−**0.84**	−0.23	
**F6**	−0.29	−**1.18**	**1.18**	−0.56	−**0.64**
**F7**	0.59	0.50	−0.37	**2.17**	**0.63**
**F8**	−0.38	**0.76**		−**1.41**	−0.47
**Proportion of Variance%**	72.0	13.9	5.9	4.2	4.0

Discriminant function analysis based on duration, source (F0) and filter (formants) variables. The covariance coefficients represent the contribution of each variable to the discrimination of the different individuals. Unsigned coefficients <0.2 are not represented. Unsigned coefficients >0.6 are bolded.

## Discussion

We identified four types of calls (three types of roars and one type of bark) in the rutting vocal repertoire of the Iberian red deer stag, defined by different combinations of duration, fundamental frequency and by the presence or absence of nonlinear phenomena (deterministic chaos). While the size of the vocal repertoire is comparable to that of other studied red deer subspecies (Scottish red deer: five call types, [30] Corsican red deer: two call types [49]), its composition is rather different.

The Long Common Roar (Figure 4) is a periodic loud call of medium duration, with a clearly defined fundamental frequency and harmonic overtones, and visible formant frequencies with downward modulation reflecting the extension of the vocal tract during phonation. While this roar is clearly homologous to the Corsican and Scottish red deer “Common Roars” [28], [30], [49], it is also characterised by the absence of nonlinear phenomena, and a relatively high fundamental frequency (180.6 Hz), which is much higher than that reported in Scottish and Corsican red deer (respectively 111.7 Hz [28] and 86.7 Hz [49]) and is in fact the highest reported amongst European red deer subspecies [28], [30], [49]. Long Common Roars were typically given at the beginning of bouts, and were longer, higher pitched, and louder than Short Common Roars.

The second type of call that we identified, the Long Common Roar with deterministic chaos, is a less frequent call characterized by the presence of chaotic segments. Common roars with deterministic chaos have also been described in Scottish and Corsican red deer [28], [49], but have not been labelled as a distinct call type. While Frey and colleagues [57] classified Iberian deer roars as either common roars (with a clearly visible F0 and harmonics) or harsh roars (without a clearly visible F0), in our sample we did not identify a distinct, entirely harsh type of roar homologous to the harsh roar reported in Scottish red deer [28], [30] and Fallow deer [38], or grunt roars (which are associated with harsh roars in red deer [28], [30]). In Scottish red deer, harsh roars are not only characterized by the absence of a clear fundamental frequency and associated harmonic overtones, but also by the virtual absence of formant modulation, reflecting the fact that the vocal tract is fully extended throughout the vocalization [28]. While Scottish red deer stags give harsh roars during intense male contests [21], [30], they also give them towards females, and it has been suggested that they may function to attract and retain female attention [43]. While relatively rare, LCRDC were identified in the vocal repertoire of 10 Iberian stags out of 13, suggesting that they are a regular feature of the mating calls of this subspecies. Interestingly, LCRDC were typically positioned at the beginning of bouts and were characterised by a relatively higher F0 and longer duration than the other Long Common Roars, suggesting that they involve a greater effort during their production. Overall the position and quality of these roars indicates that they may function to attract the attention of receivers.

We also identified a clearly distinct Short Common Roar. This roar is on average 1.48 s shorter and 50 Hz lower-pitched than the Long Common Roars. We have also measured an average difference of 10 dB in relative intensity between Short Common Roars and those of the LCR (as estimated from calls recorded in the same sequences), suggesting that SCR are produced with a lower intensity. Such calls have not been previously reported in studies of other red deer subspecies [28], [30], [49]. In Iberian deer, while Frey and Colleagues [57] report a bimodal distribution or roar duration, they do not classify SCR as a separate call type, nor contrast its acoustic properties with that of LCR [57]. The acoustic properties of these roars suggest that they may function to communicate information about size and identity over relatively short ranges.

Finally the last type of call that we identified was a series of Chase Barks, which is also given by harem holders when they chase young stags or when they herd females in both Scottish deer and Corsican red deer subspecies [28], [30], [49]. We did not however identify single barks as reported in Scottish red deer [30].

As noted by previous investigators [57], one of the key characteristics of Iberian deer roars is their relatively high F0. Despite being of a comparable size to Scottish red deer, and rather larger than Corsican deer, the F0s of Iberian stags' LCR, LCRDC and SCR are all higher than that of the Common Roar of the other subspecies [30], [49]. While investigations of F0 in the calls of Scottish red deer stags have failed to identify intraspecific correlations between fundamental frequency and body size within populations [28], males with higher minimum F0 have higher reproductive success [28], and playback experiments of resynthesized vocalisations have shown that oestrous females prefer high-pitched roars [42], suggesting that a relatively high pitched voice may be sexually selected for in this subspecies. Moreover, recent playback experiments contrasting the response of oestrous Scottish red deer hinds to homo- or hetero-specific sika deer (*Cervus nippon*) male sexual calls have shown that while red deer females typically prefer their own species vocalisations, a small proportion of individuals appear to prefer high-pitched heterospecific sika moans [12]. We suggest that the positive selection for a high F0 may reflect the fact that in mammals, with a given vocal apparatus, relatively high F0 signals can be produced more efficiently (with a greater intensity) than relatively low F0 signals [64]. Contrarily to observations by Volodin and colleagues [65], we did not identify roars with source-filter coupling phenomenon (where the F0 becomes very high and indicate a possible tuning of the source with the vocal tract resonance) in our sample.

One of the consequences of having a relatively high F0 is that it can affect the resolution of vocal tract resonances, and therefore the availability of size-related information in formant frequencies [66]. Indeed, while source (F0) and filter (formants) components can be assumed to be independently produced, the periodicity of the source signal (the glottal wave) affects the frequency spacing of the harmonics, and consequently the spectral resolution of the formant frequencies [67]. Roars delivered with a high fundamental frequency are characterised by decreased density of harmonics, and consequently by a poorer sampling of the formant envelope [19], [66]. In other words, if F0 is of a comparable magnitude, or higher than, the expected frequency spacing of the vocal tract resonances, then the glottal wave fails to provide sufficient spectral density to excite and highlight all of the vocal tract resonances. As a consequence, calls will contain little or no information on the vocal tract transfer function [45]. Previous work on perioestrus Scottish red deer hinds has suggested that a mean fundamental frequency of 130 Hz (equivalent to the mean F0 of the Iberian stag SCR) does not affect female perception of size-related formant information [19]. However the mean F0 of the LCR of Iberian red deer (180 Hz) is more than half the estimated formant spacing, which may affect the perceptibility of size-related variation in formant frequency spacing in this subspecies.

Like other red deer subspecies, Iberian red deer stags have a descended and mobile larynx enabling them to extend their vocal tract while vocalising [57]. While they tend to protrude their tongue during the majority of their roars [57], the acoustic consequences and possible functions of this gesture remain unclear [57]. When we measured formant frequencies over a short terminal section of the calls when the vocal tract is fully extended and where F0 was sufficiently low to clearly highlight vocal tract resonances, we found that the minimum formant frequencies achieved during this stage by Iberian stags were slightly higher than those reported for the (comparably sized) Scottish red deer stags [28]. The mean eVTL we estimated is slightly shorter than that reported by Frey and colleagues [57]. This discrepancy could be explained by the fact that while we measured formants during the low-pitched segments at the end of the roars, they measured formant frequencies during harsh roar segments. Indeed, taking into account only roars containing chaotic segments (LCR_DC_), when stags may put more effort into fully extending their vocal tract, ΔF and eVTL in our population (230 Hz and 76.1 cm respectively) become more similar to those reported by Frey and colleagues [57] (228.15 Hz and 76.7 cm).

Finally, the results of the discriminant function analysis confirmed the presence of individual differences, as previously identified in the loud calls of Scottish Red deer [54], Corsican deer [49] and Fallow deer [38] males. Interestingly, formant-related variables contributed strongly to discriminant functions, adding to the existing evidence that filter components play a substantial role in the acoustic distinctiveness of individuals in a wide range of mammal species (e.g. baboons (*Papio cynocephalus ursinus*): [68]; rhesus monkeys (*Macaca mulatta*): [69]; Scottish [54] and Corsican [49] red deer; elephants (*Loxodonta africana*) [70]; koalas (*Phascolarctos cinereus*) [71]; lemurs (*Eulemur rubriventer*) [72]).

In conclusion, in contrast with that of the Scottish red deer, the vocal repertoire of the Iberian red deer male is characterized by the presence of the Short Common Roar and by the lack of an entirely harsh roar. Moreover, the Long Common Roar, a call type shared with the other subspecies, shows the highest F0 observed in European deer subspecies. Our observations suggest that in Iberian red deer, sexual selection may have favoured a very high F0 in high intensity LCR, possibly at the expense of the availability of information on body size typically provided by the formant frequencies in these calls. Sexual selection also appears to have favoured the evolution of a different call type, the SCR, characterised by a lower intensity but with a lower F0 and higher spectral density, possibly enabling the communication of size and identity information through better defined formant frequencies. Playback experiments using resynthesized roars to change F0, duration, and formant frequency spacing are now needed to investigate the function of these call types and of their spectral components in both male competition and female choice contexts.

## Supporting Information

Table S1 Simultaneous Tests for Linear Hypotheses. Multiple comparisons of means with Tukey contrasts for linear mixed models. Call Type: fixed factor; Bout nested within individuals: random factors. Mean differences, standard errors (SE), t and *p* values and 95% interval of confidence for each comparison are reported.
(DOCX)
