# Supplementary material for: Roaring High and Low: Composition and Possible Functions of the Iberian Stag's Vocal Repertoire
Source: PLoS One. 2013 May 8;8(5):e63841. doi: 10.1371/journal.pone.0063841 (PMC3648515; doi:10.1371/journal.pone.0063841)
Supplement: Table S1 — Simultaneous Tests for Linear Hypotheses. Multiple comparisons of means with Tukey contrasts for linear mixed models. Call Type: fixed factor; Bout nested within individuals: random factors. Mean differences, standard errors (SE), t and p values and 95% interval of confidence for each comparison are reported. (DOCX) [file pone.0063841.s001.docx]

| **Variables** | **Comparison** | | **Mean** | **SE** | **t** | ***p*** | **95% CI** | |
| --- | --- | --- | --- | --- | --- | --- | --- | --- |
|  | **Btw Call Types** | | **Differences** |  |  |  | **Lower** | **Upper** |
| **Dur** | LCR | CB | 1.37 | 0.12 | 11.61 | <0.0001 | 1.07 | 1.66 |
|  | LCRDC | CB | 1.64 | 0.14 | 11.54 | <0.0001 | 1.29 | 2.00 |
|  | SCR | CB | -0.03 | 0.12 | -0.24 | 0.994 | -0.33 | 0.27 |
|  | LCRDC | LCR | 0.28 | 0.09 | 3.03 | 0.011 | 0.05 | 0.51 |
|  | SCR | LCR | -1.39 | 0.05 | -29.21 | <0.0001 | -1.51 | -1.27 |
|  | SCR | LCRDC | -1.67 | 0.09 | -18.53 | <0.0001 | -1.9 | -1.45 |
| **MaxF0** | LCR | CB | 73.4 | 9.1 | 8.1 | <0.0001 | 50.6 | 96.2 |
|  | LCRDC | CB | 86.5 | 10.9 | 7.95 | <0.0001 | 59.1 | 113.9 |
|  | SCR | CB | 8.6 | 9.1 | 0.94 | 0.766 | -14.3 | 31.4 |
|  | LCRDC | LCR | 13.1 | 6.9 | 1.9 | 0.207 | -4.2 | 30.5 |
|  | SCR | LCR | -64.8 | 3.6 | -18.2 | <0.0001 | -73.8 | -55.9 |
|  | SCR | LCRDC | -77.9 | 6.8 | -11.49 | <0.0001 | -95.0 | -60.9 |
| **MeanF0** | LCR | CB | 60.6 | 8.9 | 6.8 | <0.0001 | 38.2 | 83.1 |
|  | LCRDC | CB | 76.3 | 10.6 | 7.22 | <0.0001 | 49.7 | 102.9 |
|  | SCR | CB | 13.1 | 8.9 | 1.47 | 0.432 | -9.4 | 35.5 |
|  | LCRDC | LCR | 15.7 | 6.6 | 2.4 | 0.068 | -0.8 | 32.2 |
|  | SCR | LCR | -47.6 | 3.4 | -14.09 | <0.0001 | -56.1 | -39.1 |
|  | SCR | LCRDC | -63.3 | 6.5 | -9.81 | <0.0001 | -79.5 | -47.0 |
| **MinF0** | LCR | CB | 30.1 | 10.1 | 2.98 | 0.013 | 4.7 | 55.5 |
|  | LCRDC | CB | 36.7 | 11.8 | 3.1 | 0.009 | 7.0 | 66.5 |
|  | SCR | CB | 21.4 | 10.1 | 2.13 | 0.129 | -3.9 | 46.8 |
|  | LCRDC | LCR | 6.6 | 7.2 | 0.92 | 0.775 | -11.5 | 24.8 |
|  | SCR | LCR | -8.7 | 3.7 | -2.34 | 0.078 | -17.9 | 0.6 |
|  | SCR | LCRDC | -15.3 | 7.1 | -2.16 | 0.121 | -33.1 | 2.6 |
| **RangeF0** | LCR | CB | 47.2 | 9.2 | 5.14 | <0.0001 | 24.1 | 70.3 |
|  | LCRDC | CB | 54.6 | 11 | 4.97 | <0.0001 | 26.9 | 82.4 |
|  | SCR | CB | -8.9 | 9.2 | -0.97 | 0.748 | -32.1 | 14.2 |
|  | LCRDC | LCR | 7.4 | 7 | 1.06 | 0.689 | -10.1 | 25.0 |
|  | SCR | LCR | -56.2 | 3.6 | -15.56 | <0.0001 | -65.2 | -47.1 |
|  | SCR | LCRDC | -63.6 | 6.9 | -9.28 | <0.0001 | -80.8 | -46.3 |
